# Protocol for the ‘Beyond 50’ prospective observational cohort study: investigating the impact of physical and psychosocial factors on healthy ageing

**DOI:** 10.1186/s12889-025-23070-y

**Published:** 2025-11-26

**Authors:** Rose Laing, Nazgol Karimi, Tina Lam, Bosco Rowland, Paul Dietze, Aislinn Lalor, Keith Hill, Laura Alfrey, Nadine Andrew, Shalini Arunogiri, Louisa Picco, Suzanne Nielsen

**Affiliations:** 1 Monash Addiction Research Centre, Eastern Health Clinical School, Monash University, 47-49 Moorooduc Hwy, Melbourne, VIC 3199 Australia; 2 National Centre for Healthy Ageing, Melbourne, VIC Australia; 3 Faculty of Health, Deakin University, Geelong Campus, Geelong, VIC Australia; 4 National Drug Research Institute, Curtin University, Melbourne, VIC Australia; 5 Disease Elimination Program, Burnet Institute, Melbourne, VIC Australia; 6 Rehabilitation, Ageing and Independent Living (RAIL) Research Centre, School of Primary and Allied Health Care, Monash University, Frankston, VIC Australia; 7 Department of Occupational Therapy, School of Primary and Allied Health Care, Monash University, Frankston, VIC Australia; 8 Faculty of Education, Monash University – Peninsula Campus, Frankston, VIC Australia; 9 Peninsula Clinical School, School of Translational Medicine, Monash University, Frankston, VIC Australia

**Keywords:** Healthy ageing, Mental health, Physical health, Substance use disorders, Data linkage, Longitudinal cohort, Older adults

## Abstract

**Background:**

Older adults exhibit unique risks for depression and anxiety, and the current generation of 50–70-year-olds are more likely to engage in risky drinking patterns or use illicit substances than previous generations. Changing metabolism, cognition and physical health changes associated with ageing may compound effects of these behaviours. Adults aged between 50–70 also experience periods of key life transition with changes in work and family dynamics that may contribute to individuals’ ability to age healthily. This protocol paper describes the Beyond 50 Study, a prospective cohort study that aims to investigate the association between key transition periods, physical and psychosocial health, and substance use to uncover insights on healthy ageing.

**Methods:**

The Beyond 50 Study aims to recruit a cohort of 1000 adults aged 50–70 years within the Frankston and Mornington Peninsula Local Government Areas in Victoria, Australia, a region with great sociodemographic and geographic diversity. Participants will be interviewed annually using questionnaires that measure health, psychosocial and substance use domains. An ethno-epidemiological approach will be applied, to explore health and social connectedness during pivotal transition periods through in-depth interviews with a subset of participants. These complimentary interviews will inform subsequent follow up surveys. Survey data will be linked with local health data provided through the National Centre for Healthy Ageing Data Platform.

**Discussion:**

Findings from the Beyond 50 study will serve as a platform to directly inform local strategies to support healthy ageing, particularly as they relate to substance use and harm. Results will have relevance to healthy ageing in Australia and internationally.

**Supplementary Information:**

The online version contains supplementary material available at 10.1186/s12889-025-23070-y.

## Background/introduction

Between 2020 and 2050, the global population of people aged 60 and over is predicted to double to over 2.1 billion, exacerbating the total global burden of disease [1]. Adults aged 60 and over are at an increased risk of a range of adverse health outcomes, including cancer, diabetes, cognitive changes, sleep disturbance and accident and injury [2, 3]. They also exhibit unique risk factors for the development of anxiety and depression, with more than half of diagnoses occurring in later life as a result of bereavement, social isolation and periods of key life transition such as retirement [4]. While these risk factors are consistent with previous generations, research both globally and in Australia shows that individuals currently aged 50–70 exhibit increased rates of risky drinking behaviours and substance use disorders compared to previous generations [5, 6], with an estimated one in three Australian adults aged 60 and above drinking at risky levels [7]. Additionally, age-related changes in health and functioning can place individuals at an increased risk of the negative consequences (e.g. increased risk of falls) of substance use [8], which can pose new challenges for health care delivery.

Social isolation and loneliness are major public health problems which contribute to increased risk of mental health disorders such as depression and anxiety, cognitive disorders such as dementia, and death [9]. An estimated one in two people aged over 60 are at increased risk of social isolation and one third are estimated to experience loneliness in later life [10]. Furthermore, the impacts of the COVID-19 pandemic have affected how people work and socialise, with flow on effects on social isolation [11], with older adults being disproportionately affected [12].

Supporting an ageing population is a global public health challenge, and the concept of ‘healthy ageing’ has become a focus. The World Health Organization (WHO) defines healthy ageing as “the process of developing and maintaining the functional ability that enables well-being in older age” [13] with functional ability described as the ability of older individuals to meet their basic needs, build and maintain relationships, be mobile and to contribute to society [14]. Healthy ageing focuses on creating environments and opportunities that enable people to do what they value and live meaningful lives [13]. Global and Australian studies have identified many determinants of healthy ageing, including physical well-being, mental and cognitive well-being, social wellbeing, independence, community engagement, social support and financial security [15–20].

The Frankston and Mornington Peninsula local government areas are situated in the Southeast of Victoria, Australia [21, 22]. As a popular retirement destination, the Mornington Peninsula has a higher percentage of adults aged over 50 compared to the rest of Victoria (48% compared to 35% respectively) [21]. Moreover, as it is a peninsula, the majority of the population access their health and social services within these local government areas. As the population continues to age, local health services will need to ensure that they are well informed to be able to respond and support healthy ageing [23].

## Aim

The ‘Beyond 50’ study aims to follow a cohort of 1000 adults aged 50–70 years from the Frankston and Mornington Peninsula local government areas through stages of key life transition.

## Objectives

The overarching objectives of the Beyond 50 study are to:

1. Develop a detailed understanding of trajectories of physical health, mental health and substance use patterns.
2. Identify key risk and protective factors associated with the development of poor physical and mental health and substance use disorders.
3. Understand the impact of social isolation and loneliness on the development of physical, mental health and substance use disorders.

## Methods

### Study design

**Fig. 1:**
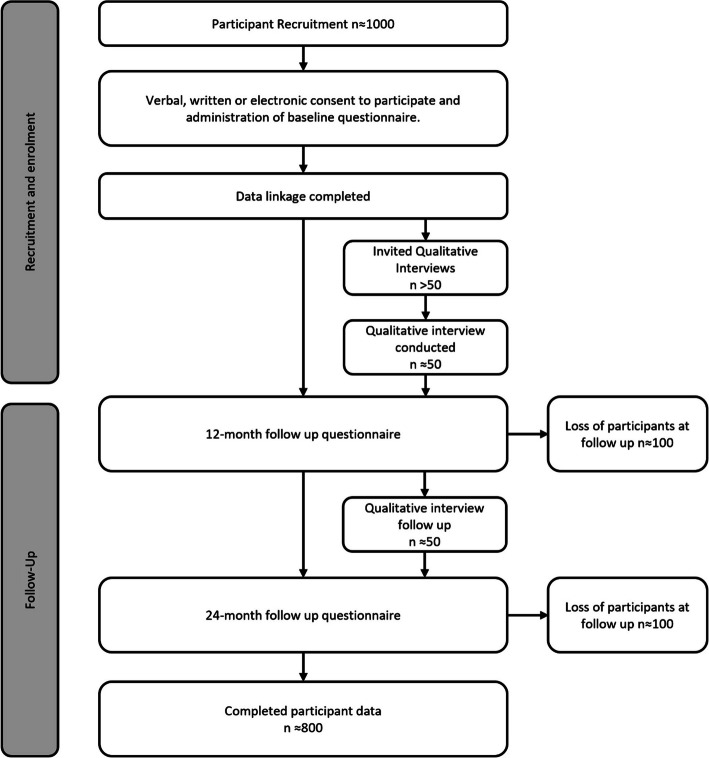
Beyond 50 prospective cohort study design

Beyond 50 is a prospective cohort study that will interview 1000 participants aged between 50–70 annually for an initial period of three years (see Fig. 1 for a flow chart overview of study design). Study outcomes will be reported according to the Strengthening the Reporting of Observational studies in Epidemiology (STROBE) checklist [24].

### Setting

**Fig. 2:**
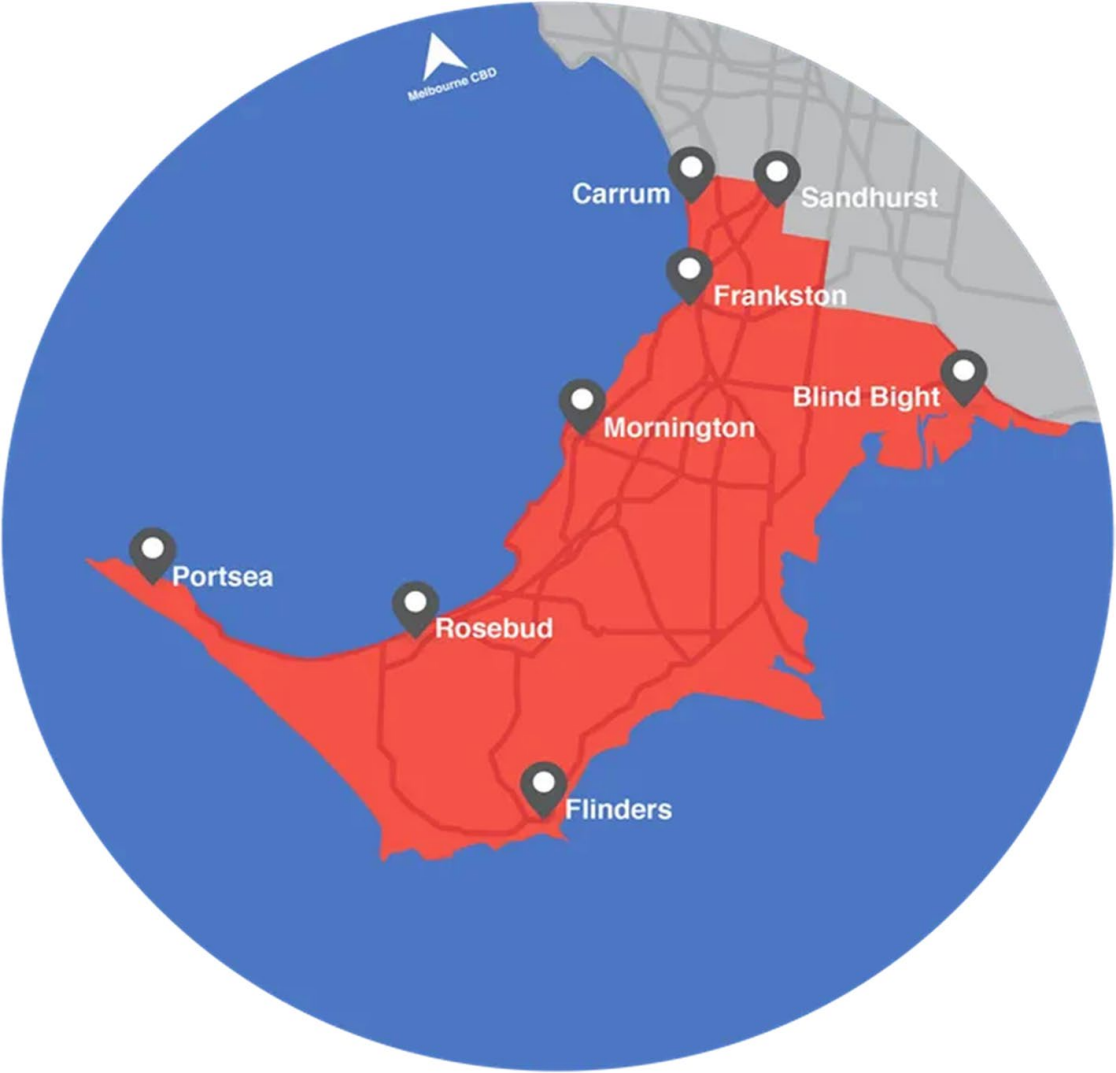
Map of frankston and mornington Peninsula local government areas

The cohort will be recruited from Frankston and the Mornington Peninsula, two adjacent local government areas within Victoria, Australia (see Fig. 2). This area has been strategically chosen for its rich geographic and socioeconomic diversity. The area has a combined population of approximately 310,000 people, with over a quarter (estimated 80,019 people) being aged 50–70 years [21, 22]. The area contains both public and private hospitals, with Peninsula Health being the sole public health provider for the region and managing four hospitals (2 acute with emergency departments and 2 subacute hospitals) and over 10 community and outpatient services, incorporating services such as mental health, community rehabilitation and palliative care. There are five private hospitals in the area, one of which has an emergency department [25, 26].

The area encompasses a geographic region that includes both metropolitan and regional areas [27], as well as great socioeconomic diversity. Based on the Australian Bureau of Statistics decile based index of relative social advantage and disadvantage (IRSAD), the region contains some of Australia’s most disadvantaged (e.g. Frankston North [Decile 1, Metropolitan], Boneo [Decile 3, inner regional]) and most advantaged (e.g. Mount Eliza [Decile 10, Metropolitan], Shoreham [Decile 10, inner regional]) suburbs [28].

### Participants

Participants must be aged 50 to 70 years at baseline survey administration and residing in either the Frankston or Mornington Peninsula local government areas, with plans to stay in the area.

### Consumer and stakeholder involvement

The Beyond 50 Study approach was developed in partnership with clinicians and local service providers, academics and members of the target audience (people aged 50–70 living in the Mornington Peninsula and Frankston local government areas) to ensure that study outcomes would be relevant for informing local service provision. A co-design consultation phase with individuals with lived experience and local service providers was undertaken to identify and develop research priorities and key aims of the study. Two workshops were conducted in February 2023, one face-to-face and one online, which were supplemented with individual interviews where participation in the workshops was not possible. Workshop participants included members from local council, Peninsula Health, the Rehabilitation, Ageing and Independent Living (RAIL) and Monash Addiction Research Centre (MARC) at Monash University, Edith Cowan University, Turning Point and the National Centre for Healthy Ageing (NCHA). Outcomes from these workshops included identifying key research priorities, key variables to measure, the age range for the proposed cohort, methods of recruitment, considerations for recruiting underrepresented groups, and the ‘Beyond 50’ study name.

Based on feedback from stakeholders and consumers, a project Advisory Board was established to provide advice and guidance to the study team in delivering on its key study aims. Members consist of 8–10 individuals with expertise in service delivery, government and non-government sector representation and individuals with lived experience. The first board meeting was held in February 2024, and it was agreed that the board meet twice per year with open correspondence in between when needed. Specifically, the Advisory Board will provide advice on recruitment strategies and approaches to aid the formation and retention of the cohort, the development of priorities for specific analyses to inform policy and practice, how to effectively translate research findings across different sectors and how to maximise the policy relevance of current and planned work.

### Recruitment

Participants will be recruited using multiple methods to minimise bias with any specific approach within the cohort. Recruitment methods include:

#### Postal mail outs

A list of deidentified addresses of individuals aged between 50–70 that live in the Frankston or Mornington Peninsula local government areas will be provided by the Australian Electoral Commission. These addresses will be randomised and mailouts containing an invitation letter addressed to the household with information on the study purpose, eligibility criteria and a link to access the survey will commence in May 2024.

#### Social media

Information about the study will be shared with local community Facebook groups through the study Facebook page, with targeted Facebook advertisements used to support recruitment.

#### Promotional flyers

Physical flyers will be distributed and displayed in key locations within the two local government areas, including community centres, medical centres, cafes, libraries and shopping centre noticeboards.

#### Website

The Beyond 50 website will contain information for participants on eligibility, enrolment, and study updates. An expression of interest form will be included to allow for eligibility screening.

#### Study promotion via local networks

Stakeholders from the local area, including those involved in the initial co-design process will assist with promotion through word-of-mouth, distribution of study flyers and promotional information and suggestions of new methods of recruitment.

### Data collection

At study outset, three annual ‘waves’ of data collection are planned. Potential participants will be provided with a participant information sheet detailing the purposes and procedures of the study, including the various methods in which participation can occur (i.e. self-administered online, phone interview or face-to-face). Prior to data collection commencing, participants will need to provide informed consent for participation in the study. Participants will be followed up at 12- and 24-months post completion of baseline survey, via their preferred method of contact and invited to complete follow up interviews via their preferred mode of delivery. Upon completion of each survey, eligible participants will receive a AUD40 gift voucher, with all data collected being stored in a de-identified manner. All contact details and identifying information is stored separately on secure servers.

Study data will be collected and managed using REDCap (Research Electronic Data Capture) [29] and hosted and managed by Helix (Monash University).

### Measures for quantitative interviews

An overview of instruments used within the quantitative survey are outlined in Table 1, and details on scoring and validation can be seen in *Additional file 1.*

**Table 1:** Measures and Instruments used in survey

**Measures**	**Instruments**	**Information captured**	Baseline	12-Month follow-up	24-Month follow-up
**Introduction/Screening**		Consent and contact details (phone number and email address)	**✓**		
**Demographics and socioeconomic status**	Individual items from Australian Bureau of Statistics/Australian Institute of Health and Welfare [30, 31]	Demographics, as well as work, socioeconomic status, geographical location and relationship status	**✓**	**✓**	**✓**
**Quality of Life**	12-Item Short Form Survey Instrument (SF-12) [32]	Physical and mental health	**✓**	**✓**	**✓**
**Co-morbidities**	Self-Administered Co-Morbidity Questionnaire [33]	Presence of comorbidities, if treatment is being received for them and whether they interfere with daily function	**✓**	**✓**	**✓**
**Service Utilisation**	Healthcare utilisation (6 items)	Unstandardized questions about the frequency of health service utilisation over last 12 months	**✓**	**✓**	**✓**
**Functional Status**	Functional Status Questionnaire (FSQ) [34]	Occupational and social role and function over the last month	**✓**	**✓**	**✓**
**Social Support**	Duke Social Support Index (DSSI) [35]	Social support and satisfaction with social interaction	**✓**	**✓**	**✓**
**Loneliness**	University of California-Los Angeles Loneliness Scale 4-item version (UCLA – 4) [36]	Measure of perceived loneliness	**✓**	**✓**	**✓**
**Anxiety**	General Anxiety Disorder Assessment (GAD-7) [37]	Symptoms of anxiety over last 2 weeks	**✓**	**✓**	**✓**
**Depression**	Patient Health Questionnaire (PHQ-9) [38]	Symptoms of depression over last 2 weeks	**✓**	**✓**	**✓**
**Cannabis Use**	The Cannabis Use Disorder Identification Test Short Form (CUDIT-SF) [39]	Current cannabis use and severity of potential cannabis use disorder	**✓**	**✓**	**✓**
**Other Drug Use**	Alcohol, Smoking and Substance Involvement Screening Test-lite version (ASSIST-lite) [40]	Use of other substances both illicit and prescribed	**✓**	**✓**	**✓**
**Alcohol Use**	Alcohol Use Disorders Identification Test-Consumption (AUDIT-C) [41]	Frequency of alcohol consumption and severity of potential alcohol use disorder	**✓**	**✓**	**✓**
**Gambling Behaviours**	Household, Income and Labour Dynamics in Australia (HILDA) [42]	Amount, in Australian dollars, spent gambling on different activities in a typical month	**✓**	**✓**	**✓**
	Problem Gambling Severity Index (PGSI)- mini screen [43]	Severity of gambling behaviours	**✓**	**✓**	**✓**
**Childhood Experiences**	Positive Childhood Experiences (PCE-7 items) [44]	Positive childhood experiences, including support and communication	**✓**		
	Difficult Childhood Questionnaire (DCQ- 3)() [45]	Negative childhood experiences, including family conflict and childhood trauma	**✓**		
**Food Security**	Food security (6 item food security) [46, 47]	Food security over last 6 months	**✓**	**✓**	**✓**
**Financial Stress**	Financial Stress-(Australian Bureau of Statistics) (9-item) [48]	Financial stress over last 12 months	**✓**	**✓**	**✓**
**Pain**	Pain, Enjoyment of Life and General Activity scale (PEG −3) [49]	Pain severity and interference with everyday life over last week	**✓**	**✓**	**✓**
**Physical Activity**	International Physical Activity Questionnaires (IPAQ) [50]	Frequency and level of physical activity over last 7 days	**✓**	**✓**	**✓**
**Insomnia**	Insomnia Severity Index (ISI) [51]	Severity of current insomnia problems over last 2 weeks	**✓**	**✓**	**✓**
**Locator Form**		Contact details and contact preferences	**✓**		
**Data Linkage**		Details needed for data linkage	**✓**		

#### Demographics and socioeconomic status

Measures include date of birth, gender, sexual identity, relationship status, work and retirement status, household income and highest level of education attained, with items taken from within the Australian Bureau of Statistics and Australian Institute of Health and Welfare surveys [30, 31].

#### Quality of life

Will be measured using the 12-Item Short Form Survey Instrument (SF-12) [32].

#### Physical health

Comorbidities will be measured using The Self-administered Co-Morbidity Questionnaire (SCQ) [33, 52]. Medical service utilisation including general practitioner (GP) appointments, ambulance services, emergency department and inpatient/outpatient visits over the previous 12 months will be measured using a 6-Item Healthcare Utilisation questionnaire. Functional Status will be measured using The Functional Status Questionnaire [34].

#### Mental health

Will be measured using the 7-item General Anxiety Disorder Assessment (GAD-7) [37] and the 9-item Patient Health Questionnaire-9 (PHQ-9) [38].

#### Social support and loneliness

Social support will be measured using the 11-item Duke Social Support Index [35], while loneliness with be measured using the 4-item Los Angeles Loneliness Scale (UCLA-4) [36].

#### Substance use

Will be measured using the Cannabis Use Disorder Identification Test Short Form (CUDIT-SF) [39], Alcohol, Smoking and Substance Involvement Screening Test (ASSIST-lite) [40] and Alcohol Use Disorders Identification Test (AUDIT-C) [41, 53].

#### Gambling Behaviours

Will be measured using the Problem Gambling Severity Index (PGSI-mini screen) [43] with one additional item from the Household, Income and Labour Dynamics in Australia (HILDA) Study Questionnaire (QC6) [42].

#### Childhood experiences

Will be measured using the Positive Childhood Experiences (PCE-7 items) [44] and a 3-item Difficult Childhood Questionnaire (DCQ-3) [45].

#### Food and financial security

Will be measured using the 6-Item Food Security survey [46, 47] and nine Financial Stress Indicators from the Australian Bureau of Statistics Household Expenditure Survey [48].

#### Pain

Will be measured using the Pain, Enjoyment of life, and General activity survey (PEG-3) [49].

#### Physical activity

Will be measured using the International Physical Activity Questionnaires (IPAQ) [50, 54].

#### Insomnia

Will be measured using the Insomnia Severity Index (ISI) [51].

#### Contact and data linkage details

Contact details will be collected post survey completion. Personal information will be verified via a phone call after the completion of the survey, to ensure accuracy for data linkage processes.

### Cohort maintenance

To support cohort retention, a locator form will be used to collect contact information including primary and secondary contact details, preferred method of contact and preferred time of contact, upon completion of the baseline survey and updated at each ‘wave’. Details will be stored in a second secure database separate from survey responses.

Additional maintenance methods will include personal communication with participants, with each participant talking directly to a study team member at least once post baseline. Twice a year Beyond 50 newsletters will be distributed to keep participants up to date on study findings, to maintain interest and promote continuation and provide participants an opportunity to contact the study team to update their contact details.

### Data-linkage

The Frankston and Mornington Peninsula local government areas offer the unique advantage of enabling data linkage through the NHCA Healthy Ageing Data Platform, which provides comprehensive healthcare data from all public hospitals, community health and outpatient clinics provided by Peninsula Health [55]. Data from over 11 different datasets are brought together, internally linked within the Peninsula Health data centre and is updated on a weekly basis. Environmental data such as walkability, greenery and parks data has also been incorporated into the Platform. Data linkage will provide up to 10 years of historical data prior to the participants enrolment and a minimum of five years for prospective health outcome data [56], with the potential to extend this into the future pending further funding.

Data obtained through interviews will be linked to participants’ de-identified health data through the NCHA Data Platform, for all attendances to Peninsula Health run services (inpatient and community based). A two-stage, privacy preserving data linkage model will be used in which identifying data in the form of full name, residential address, date of birth, gender and date of death (if applicable) will be extracted from the cohort study contact details database and submitted to the NCHA Data Operations Manager. Probabilistic linkage software will be used to match identifying information from the cohort with the same identifiers held within the NCHA data platform, housed within the Peninsula Health Data Centre. A linkage key (or linkage ID) will be generated for each matched participant and attached to the de-identified records held within the Data Platform. For cases where a 100% probable match is not achieved, a manual review will take place to verify the match. If a case is still uncertain after manual review, the participant will be contacted to verify the match. This will ensure that there are no false positive matches within the dataset. A linkage map, in which the linkage key is mapped to the project ID, will be created and provided to the researcher team so that each individuals’ cohort data can be merged with their de-identified healthcare data. The de-identified data will be transferred to Monash University’s Secure eResearch Platform (SeRP) for storage and analysis.

### Sample size calculations

A baseline sample of 1000 participants will be collected and will allow an attrition of 200 people over the life of the project. Sample size calculations were determined based on the ability to detect the difference between high and low levels of social support and loneliness within the cohort. Australian data suggest that approximately 39% of individuals over 50 with depressive symptoms (PHQ-9 m = 5.2) and 19% of individuals without depressive symptoms (PHQ-9 m = 3.2) have low levels of social support [56, 57]**.** Power calculations using Stata power two proportion command indicates that with an 80% retention of the original sample (*n* = 800), a significance level of 0.05, the study has > 95% power to detect this difference in depression between those with high and low levels of social support at each wave. Moreover, with an alpha of 0.05, assuming an ICC of 0.05 within clinics in the four hospitals and an ICC of 0.01 between individuals in clinics, a correlation of the PHQ-9 over two timepoints as 0.56, a SD of 4 for the PHQ-9 for the whole sample, and an attrition of 200 (*n* = 800), we have 84% power to detect a minimal clinical important difference (MCID) in the PHQ-9 of 1.3 between groups over two time points [57]**.** This will be assessed over time by group interaction between lonely and not lonely respondents between baseline and a subsequent wave. Power estimations calculated by General Linear Mixed Model Power and Sample Size (GLIMMPSE) software.

### Data integrity

Several preventative and corrective measures will be implemented to ensure integrity of the data set and to minimise non-genuine responses [58]. Preventative measures will include use of CAPTCHA, collection of phone number and email address at the start of the baseline survey and not sharing direct survey links on social media platforms. Corrective measures will include screening and validation of collected data through manual checks and the establishment of electronic reports to identify patterns in response rates that may be non-genuine. Responses identified as potentially non-genuine will be flagged as suspicious and excluded from analyses if validity of responses is unable to be confirmed (i.e. if participants not able to be contacted directly to verify eligibility).

### Ethno-epidemiology sub study

For this phase, a subset of participants, specifically those entering significant transition periods (e.g., retirement), will be invited to take part in two in-depth qualitative interviews: one at the outset of data collection and another 12 months later (see Fig. 1). After providing a separate informed consent for participation, semi-structured interviews will be conducted with a focus of delving deeper and examining the relationship between life stage transition, social isolation and loneliness and health outcomes following an interview schedule (*see Additional file 2)*. Interviews will be conducted via the participants preferred method (phone, online or face-to-face) with verbal or written consent required before the interview proceeds.

Recruitment will proceed iteratively until enough information power is achieved, emphasizing data richness and depth to generate meaningful themes through reflexive thematic analysis, with an anticipated 50 participants to be recruited.

Interviews will be audio-recorded and will be transcribed verbatim by a professional transcription service, with each transcript crosschecked for accuracy by interviewers. Participants will receive a AUD40 gift voucher on interview completion. Several steps will be taken to remove bias, such as emphasizing to interviewees that the researcher is solely interested in their honest thoughts and perspectives, with no right or wrong answers and assuring interviewees that no identifying information will be published.

### Analysis

#### Quantitative data analysis

Descriptive statistics will be used to determine the prevalence of key variables within the cohort. Parametric and non-parametric statistical analysis techniques will be used to examine the correlation between key variables and health outcomes. Generalised estimation equations (GEE) and other statistical techniques ideally suited to correlated data will be used to examine the extent to which different variables and risk factors are associated with later health outcomes. As count data outcomes such as emergency department admissions are likely to have many zero counts and skewed distribution of non-zero counts, negative binomial or zero-inflated negative binomial analysis, or Poisson or zero inflated Poisson depending on if there is over dispersion of data, approaches will be used. Survival/competing survival analysis will be used to examine risk of and time to key health outcomes. GEE and survival analysis will control for a range of confounding factors identified at baseline and at follow-ups. Growth modelling will be used to identify constant and dynamic influences that affect the initiation of health behaviours (intercept trajectory) and the rate (slope of trajectory) of behaviour change over time. This will allow for incorporation of different influences at key ages and stages in the developmental life course of the cohort.

#### Qualitative data analysis

Interview transcripts will undergo thematic analysis, following the phases outlined by Braun and Clarke [59]. This process entails familiarising oneself with the data, coding it, generating initial themes, developing and reviewing themes, refining, defining and naming themes, before the final write-up. An inductive approach will be employed to allow themes to be derived through a data-driven exploration of the dataset. Three interviewers will utilise reflexivity within the inductive thematic analysis, critically reflecting on their own role as researchers within the research process itself. Interviewers will immerse themselves in the interview transcripts to identify themes that emerge naturally from the participants’ interview data. Open discussions within the research team will be undertaken during the thematic development phase. Themes will be actively produced through the systematic engagement of the researchers with the data. Participants’ quotations with some non-identifiable characteristics like gender and age will be used to illustrate the themes, but owing to ethical considerations, after the second interview, no further contact will be made with participants to obtain more feedback.

NVIVO software will be used to facilitate different stages of data analysis. To enhance methodological rigor and transparency, the Consolidated Criteria for Reporting Qualitative Research (COREQ) checklist [60] will be followed in presenting the methods and results.

The qualitative sub-study will provide a unique longitudinal dataset that complements the quantitative data, enriching and guiding future waves of quantitative data collection. As quantitative data identifies participants for the qualitative sub-study, the themes emerging from qualitative interviews will directly inform the direction of quantitative analysis. This iterative process will suggest additional measures for inclusion in subsequent data collection waves, fostering a holistic understanding of participants'experiences.

## Discussion

### Project outcomes

The Beyond 50 study aims to provide new knowledge of risk and protective factors during times of key life transition, to identify and understand how different health and social factors contribute to the development of anxiety, depression and substance use disorders. An ageing population who exhibits unique risk factors for physical and mental health and substance use disorders poses new challenges to healthcare provision. Given the high proportion of older adults in the Frankston and Mornington Peninsula local government areas, this study is ideally positioned to address existing gaps to better understand the association between different health and social factors and healthy ageing. The results of this study can have local and international relevance in understanding how health and social factors contribute to the development of physical and mental health and substance use disorders.

### Strengths and limitations

Strengths of the Beyond 50 cohort study include the diversity of the Frankston and Mornington Peninsula local government areas, which encompasses urban and regional areas with considerable socioeconomic and geographic diversity. This diversity will result in data that represents a broad range of the population and can contribute to an understanding of how physical and mental health and substance use disorders develop across a diverse population. The study includes a range of measures capturing physical health, mental health and substance use, many of which have been validated in older adult populations, providing the opportunity to explore a range of risk and protective factors associated with healthy ageing. The ethno-epi approach being adopted in the qualitative interviews will allow for in-depth understanding and provide a unique insight into individuals’ experiences during periods if key life transition. Data linkage capturing hospital attendances and health outcomes will complement direct data collection over multiple domains including physical and mental health, psychosocial and substance use domains, and will provide a rich understanding of the experiences and outcomes for this cohort.

Although outcomes measured through linked data are not subject to bias due to attrition, attrition is common with longitudinal data collection. To minimise attrition, several cohort maintenance strategies will be implemented including collection of locator form details, personal communication with all participants via phone calls and twice-yearly distribution of newsletters to keep participants updated with study developments. As non-genuine responses can bias results if included in data analysis, the study utilises a robust quality control and data check protocol including both risk minimisation and elimination methodologies, and the likelihood of non-genuine responses being included in the final dataset is extremely low [58]. Incomplete capture of service utilisation may occur as the NCHA Data Platform only captures data for the public health system and not for the private hospitals in the region, one of which contains an emergency department. Peninsula Health reported 98,168 emergency department presentations between 2022–2023 and represents one of the busiest emergency departments in Victoria [25], and while information for emergency utilisation for private emergency departments are not publicly available, the private emergency department in the area has only 14 bays and is expected to represent a very small number of emergency visits compared to the local public health system. Information relating to private emergency department and hospital access will be captured during quantitative data collection (see Table 1) which will mitigate this potential loss of information and allow us to assess the extent to which presentations at these services are missed.

## Ethics and dissemination

The study was approved by the Monash University Human Ethics Committee (#96756) and Peninsula Health Human Ethics Committees (#35909). Findings will be disseminated at local and international conferences and peer reviews journals, as well as via local stakeholder presentations and other relevant dissemination approaches. Dissemination of results to key stakeholders can inform local healthcare providers and policymakers in developing evidence-based strategies to support healthy ageing and develop mental health and substance use disorder prevention strategies.

## Supplementary Information

Additional file 1. Table of Validated Instruments Used in Quantitative Surveys and Calculation of Scores. Contains a table of the validated instruments and scales measured in baseline data collection in the beyond 50 study.

Additional file 2. Qualitative Interview Guide. Health and Social Outcomes in Frankston and the Mornington Peninsula Qualitative interview guide for the Beyond 50 study baseline data collection.

## Data Availability

No datasets were generated or analysed during the current study.

## Abbreviation

NCHA: National Centre for Healthy Ageing
