# Supplementary material for: Protocol for the ‘Beyond 50’ prospective observational cohort study: investigating the impact of physical and psychosocial factors on healthy ageing
Source: BMC Public Health. 2025 Nov 26;25:4157. doi: 10.1186/s12889-025-23070-y (PMC12659142; doi:10.1186/s12889-025-23070-y)
Supplement: Supplementary file 1 — Additional file 1. Table of Validated Instruments Used in Quantitative Surveys and Calculation of Scores. Contains a table of the validated instruments and scales measured in baseline data collection in the beyond 50 study. [file 12889_2025_23070_MOESM1_ESM.pdf]

Table of Validated Instruments Used in Quantitative Surveys and Calculation of Scores

Instrument	Measure	Calculation/ Validation
12-Item Short Form Health Survey Instrument (SF-12)	Quality of Life	This shortened version of the 36-item Short Form Health Survey (SF-36) is considered reliable in clinical and research settings. Scores are calculated from 12 items covering various aspects of physical and mental health. Responses are weighted and scored using a specific algorithm to generate two summary scores (physical component summary and mental component summary). Scores are standardized with a mean of 50 and a SD of 10 in the general population, with scores above 50 indicating better than average health, while scores below 50 indicate lower than average health.
The Self-Administered Co-Morbidity questionnaire (SCQ)	Chronic Health Conditions	Includes a total of 13 chronic health conditions, with an option to self-report other chronic health conditions. Participants are able to select all response options that are applicable to them. This is proven to be an efficient method to assess chronic health conditions and comorbidities and is particularly useful in research where medical records are unavailable
The Functional Status Questionnaire	Participants' function at work, and social function	The 6-item occupational function survey to measure participants' function at work, and three individual items that were selected to measure social function. Questions include how many days in the last month an illness or injury had kept the participant in bed for either all or most of the day, how many days in the last month did the participant cut down on the things they usually do for a half-day or more due to an illness or injury, and satisfaction with sexual relationships
Duke Social Support Index (DSSI)	Satisfaction with social support and social interaction	A 10-item measure summed and scored between 11-33, where higher scores indicate higher levels of social support. Up to two missing values can be imputed as the mean of the remaining values to create DSSI score<br>Subscales: Items 1-4 are scored on the social interaction scale (ranges from 4 to 12), and items 5 to 11 are scored on the subjective support scale (ranges from 7 to 21). Missing values cannot be imputed for subscales
University of California, Los Angeles Loneliness Scale (UCLA-4)	Loneliness	A 4-item measure recorded on a 4-point Likert scale, summed to a score of 4-16, where higher scores indicate higher levels of subjective loneliness
General Anxiety Disorder Assessment (GAD-7)	Anxiety	A 7-item measure recorded on a 4-point Likert scale. Items are coded and summed to a score between 0-21, where higher scores indicate greater severity of symptoms of anxiety. A score of 0-4 indicates minimal anxiety, 5-9 indicates mild anxiety, 10-14 indicates moderate anxiety and 15-21 indicates severe anxiety
Patient Health Questionnaire-9 (PHQ-9)	Depression	A 9-item measure recorded on a 4-point Likert scale. Items are coded and summed to a score between 0-27, where higher scores indicate greater severity of symptoms of depression. A score of 0-4 indicates minimal depression, 5-9 indicates mild depression, 10-14 indicates moderate depression, 15-19 indicates moderately/severe depression and ≥20 indicates severe depression
Cannabis Use Disorder Identification Test Short Form (CUDIT-SF)	Current cannabis usage	A 3-item measure recorded on a 4-point Likert scale. Items are summed to a score of 0-12, where higher scores indicate greater severity of cannabis use disorder. A score of 2 or higher is considered a positive screen for cannabis use disorder
Alcohol, Smoking and Substance Involvement Screening Test (ASSIST-lite)	Use of tobacco, stimulants, sedatives and psychedelics	Each substance consists of 3 questions with binary response options (Yes or No). Items are summed and scored between 0-3, with higher scores indicating higher risk of substance use disorder. A score of 0 indicates low risk, 1-2 indicates moderate risk and 3 indicates high risk
Alcohol Use Disorders Identification Test (AUDIT-C)	Risk of hazardous alcohol consumption	A 3-item measure summed to a score of 0-12, where higher scores indicate greater likelihood that alcohol consumption is at level hazardous to health. A score of 0 indicates no alcohol use, and a score or 4 for men or a score of 3 or more for woman is considered a positive screen for hazardous drinking. AUDIT-C has been validated as a highly accurate indicator of alcohol use disorder in older adults
Household, Income and Labour Dynamics in Australia (HILDA) Study Questionnaire	Amount of AUD spent on gambling activities in atypical month	Question C6 of the HILDA Questionnaire. Participants indicate how much (in AUD) they spend on gambling activities in a typical month.
Problem Gambling Severity Index (PGSI-mini screen)	Gambling behaviour and consequences	A 3-item measure recorded on a 4-point Likert scale. Items are summed to a score of 0-9, where higher score indicates higher risk of problematic gambling. A score of 0 indicates non-problem gambler, 1 indicates low-risk gambler, 2-3 indicates moderate risk gambler and 4< indicates problem gambler
Positive Childhood Experiences (PCE-7 items)	Positive Childhood Experiences	A 7-item measure recorded on a 5-point Likert scale. Items are summed to a score of 0-7, where higher scores are associated with a greater amount of positive childhood experiences.
Difficult Childhood Questionnaire (DCQ-3)	Adverse Childhood Experiences	A 3-items measure recorded on a 5-point Likert scale. Items are summed and scored, with higher scores indicating greater perceived difficulties in childhood

6-Item Food Security survey	Food security	A 6-item measure that is summed to a score of 0-6, where higher scores indicate lower food security. A score of 0-1 indicates high or marginal food security, 2-4 indicates low food security and 5-6 indicates very low food security
Australian Bureau of Statistics Household Expenditure Survey	Financial Stress	A 9-item measure where each items response is recorded as 'Yes', 'No' or 'Not applicable'
Pain, Enjoyment of Life and General Activity scale (PEG-3)	Pain Intensity	A 3-item measure where each item is recorded on a scale of 0-10, and higher scores indicate greater pain severity. A score of 0 indicates no pain and 10 indicates highest imaginable pain. The mean of the scores from the 3 items is used as the final score
International Physical Activity Questionnaires (IPAQ)	Frequency and level of physical activity	Participants are asked to report how often in the last week they have completed at least 10 minutes of four different categories of physical activity, and for how long those activities were calculated. Computation of the final score requires summation of duration of activity and frequency at which it is performed
The Insomnia Severity Index (ISI)	Perception of insomnia severity	A 7-item measure where items are summed to a score of 0-28. Higher scores indicate higher levels of insomnia
