# Supplementary material for: Protocol for the ‘Beyond 50’ prospective observational cohort study: investigating the impact of physical and psychosocial factors on healthy ageing
Source: BMC Public Health. 2025 Nov 26;25:4157. doi: 10.1186/s12889-025-23070-y (PMC12659142; doi:10.1186/s12889-025-23070-y)
Supplement: Supplementary file 2 — Additional file 2. Qualitative Interview Guide. Health and Social Outcomes in Frankston and the Mornington Peninsula Qualitative interview guide for the Beyond 50 study baseline data collection. [file 12889_2025_23070_MOESM2_ESM.pdf]

Qualitative Interview Guide. Health and Social Outcomes in Frankston and the Mornington Peninsula

Health and social outcomes in the Frankston and Mornington Peninsula (Qualitative Interview Guide) Version 1.0 (30.03.2023)

This qualitative research interview aims to explore the experiences changes in health and social worlds for people who are 50-70. The goal of this interview is to gain an in-depth understanding of how you perceive and navigate changes over this time, including the physical, psychological, and social aspects of health.

If employed:

Firstly, can you tell me about your current job? Are you working?

Do you have an anticipated date of retirement?

What are your reasons for retirement? (Is it voluntary)?

What do you believe the next steps out of the workforce will look like and be for you? Examples on changes on relationships, lifestyle, time management.

If retired

Have you retired? If so, how long ago was that?

Was retirement planned or unplanned?

Did your transition out of the workforce occur as you anticipated it would go?

What supported your readjustment after leaving the workforce?

What help did you need that wasn't available?

Can you tell me a little more about what this was like for you?

What was it like to retire? What changed? What didn't change?

Social World

Who do you live with (now and when in workforce)?

How, if at all, are your social connections changing?

How, if at all, are your family relationships changing?

How, if at all, are your relationship with your partner changing?

What helps you maintain social connections?

Do you have people that you can reach out to and ask for help at any time?

What community or other groups are you involved with? How often do you meet?

Do you experience any barriers to attending or engaging with these community or other groups?

What activities would you like to have access to?

What barriers have you experienced when endeavouring to access activities that you would like to be involved in?

What has worked well for you and why?

Did you notice changes in your use of alcohol, any other medicines (prescribed or not), or other substances during or after the transition from employment to retirement?

If experienced social isolation

Can you describe what social isolation looks or feels like for you?

What social or community groups would you have liked to have access to or have known about (e.g. when leaving the workforce)?

What social or community groups would you like to have access to now?

Did your experiences of social isolation impact your health and wellbeing? Can you give me some examples?

What impact, if any, has social isolation had on your use of alcohol, any other medicines (prescribed or not), or other substances?

What difference, if any, do you think access and knowledge of technology makes in relation to social connections?

Do you drive/have access to a motor vehicle?

How would you describe your mobility/physical health?

If did not experience social isolation

Were there any changes in your health and wellbeing after this transition out of the workforce?

Has your social world changed at all? If so, how? If not, why not?

What is the greatest strength/relationship in your social world?

What challenges/barriers have you observed in peers who experience social isolation?

Do you have access to a car/technology/community support service?

How would you describe your mobility/physical health?

Loneliness

Who do you live with?

What community/home help services are you engaged with?

How, if at all, did your transition out of the workforce relate impact loneliness?

Do you feel a sense of belonging in/to a local community?

Can you describe what loneliness looks and feels like for you?

What would a satisfying relationship look like to you?

Do you believe there is stigma attached to loneliness? If yes, what does this look like? (i.e., personal failure)

Is loneliness experienced at certain times of the day or week, or after certain events?

Does groups or activities for 'old' or 'senior' people act as a barrier to you participating (i.e., not identifying with these groups)

If experienced loneliness

How have your experiences of loneliness impacted your health and wellbeing?

When do you feel lonely?

Are there times you do not feel lonely? Can you give an example?

If did not experience loneliness …

Were there times you did feel lonely? Give an example.

At these times what stopped your loneliness?

Health Outcomes

How, if at all, did changes in your social world impact your health and wellbeing?

How, if at all, have changes in your social world (e.g. transition out of the workforce) related to your experiences of health and wellbeing?

Were there any changes in your mental health after this transition? If so, what were they? If not, why not?

What challenges did you experience in regard to your mental health?

What information wasn't available during this time that you wished you had?

What services would you have liked access to during this time?

What do you think may have helped with challenges you faced?

Did your health priorities change after retirement? Or What are your health priorities at present?
